# NON-INVASIVE BLOOD PRESSURE AND OTHER PHYSIOLOGICAL DATA IN CHEMICALLY IMMOBILIZED BROWN BEARS (*URSUS ARCTOS*)

**DOI:** 10.1016/j.dib.2020.105646

**Published:** 2020-04-29

**Authors:** Jacopo Morelli, Angela Briganti, Boris Fuchs, Ðuro Huber, Alina L. Evans, Natarsha Babic, Slaven Reljić, Lana Pađen, Jon M. Arnemo

**Affiliations:** a Department of Forestry and Wildlife Management, Inland Norway University of Applied Sciences, 2480 Koppang, Norway; b Department of Veterinary Sciences, University of Pisa, 56122 San Piero A Grado, Italy; c Department of Biology, Faculty of Veterinary Medicine, University of Zagreb, 10000 Zagreb, Croatia; d Department of Physiology, Faculty of Veterinary Medicine, University of Zagreb, 10000 Zagreb, Croatia; e Department of Wildlife, Fish and Environmental Studies, Swedish University of Agricultural Sciences, 901 83 Umeå, Sweden

**Keywords:** Brown bear, Blood pressure, Korotkoff, Hypertension, Heart rate, Respiratory rate, Temperature, Capture, BP, blood pressure, HR, heart rate, RR, respiratory rate, SpO_2_, hemoglobin-oxygen saturation, Tr, rectal temperature, SAP, systolic arterial blood pressure, DAP, diastolic arterial blood pressure, MAP, mean arterial blood pressure, SD, Standard Deviation, CW/LC, cuff width/limb circumference, Ta, ambient temperature, XK, xylazine-ketamine, IM, intramuscularly, GPS, Global Positioning System, MTZ, medetomidine-tiletamine-zolazepam, VHF, Very High Frequency

## Abstract

Free-ranging brown bears (*Ursus arctos*) were snared and subsequently darted with a combination of xylazine-ketamine in Croatia (n = 5) or darted from a helicopter with a combination of medetomidine-tiletamine-zolazepam in Scandinavia (n = 20). Three adults and one yearling (1 year old) bear were captured in Croatia, with one adult being captured twice. The Scandinavian bears were divided into Group A (yearlings, n = 7) and Group B (subadults, n = 2 and adults, n = 11). The exertion time (time from activation of the trap or from the start of the helicopter chase to recumbency) and the induction time (time from darting to recumbency) were recorded. The rectal temperature (Tr) was measured as soon as possible after induction and then monitored at frequent intervals (varied between individuals) in immobilized bears. Blood pressure (BP) was measured with a non-invasive method (Korotkoff's technique) every 5 minutes. The heart rate (HR), respiratory rate (RR), and arterial haemoglobin oxygen saturation (SpO_2_) were recorded every 5 minutes. Reliability of the BP monitoring technique, trends of variation of the physiological variables, and the factors related to the capture were assessed. Both exertion and induction times were longer in Croatian bears than in Scandinavian bears. In Croatian bears, the Tr was either constant or slightly decreasing, with hyperthermia recorded in two individuals (Tr > 39.0° C). In Scandinavian bears, 17 of 20 individuals developed an initial hyperthermia. Four of five bears in Croatia and 17 of 20 bears in Scandinavia showed a decreasing trend in systolic and mean BP over time. According to the Korotkoff method, all bears were hypertensive (mean BP > 130 mmHg) with varying severity, and the systolic pressure was significantly lower in yearlings when compared to subadults and adults. Yearlings had significantly (p < 0.05) higher HR than subadults and adults, however there was no significant differences in RR, SpO_2_, and Tr between the age groups. All Croatian bears and 13 of 20 Scandinavian bears were moderately to severely hypoxemic (SpO_2_ < 90%). Further studies with simultaneous invasive and non-invasive (Korotkoff) BP monitoring techniques are required to confirm the accuracy of methods used in this study. The data presented here provides evidence of the physiological impact of different capture methods and chemical immobilization of brown bears in Croatia and Scandinavia.

**Specifications Table**

**Table:** 

**Subject**	Veterinary Science
**Specific subject area**	Wildlife physiology and medicine
**Type of data**	Table Figure
**How data was acquired**	Pulse-oximeters (OxyVet, Eickemeyer Veterinary Equipment Ltd, and NPB-40, Infiniti Medical®, used in Croatia and in Scandinavia, respectively), stethoscope (Littmann® Classic II S.E., 3M®), digital thermometer (Wellkang Ltd), standard aneroid sphygmomanometer (DM330LF, LOGIKO VISUAL®, Moretti S.p.a.).
**Data format**	Raw Analyzed
**Parameters for data collection**	Five bears were snared and darted with xylazine-ketamine in Croatia and 20 bears were darted from a helicopter with medetomidine-tiletamine-zolazepam in Scandinavia. Physiological parameters were measured with either the same or similar instruments in Croatia and Scandinavia, and the impacts of the different capture method and drugs on the physiology of bears were assessed.
**Description of data collection**	The exertion time (time from the activation of the trap or from the start of the helicopter chase to recumbency) and the induction time (time from darting to recumbency) were recorded at the time of occurrence. Heart rate (HR), respiratory rate (RR) and hemoglobin-oxygen saturation (SpO_2_) were measured by pulse oximetry and a stethoscope every 5 minutes or at frequent intervals (varied between individuals). Rectal temperature (Tr) was measured with a digital thermometer inserted 10 cm deep into the rectum at frequent intervals (varied between individuals). The hair proximal to either the carpus or the tarsus was clipped, the limb circumference was measured, and a cuff of appropriate size was placed. The manual technique detected Korotkoff's sounds of the pulse through the diaphragm of a stethoscope placed above the medial plantar artery, distally from the cuff of the sphygmomanometer. Systolic (SAP) and diastolic (DAP) blood pressure were measured in order to obtain from one to three values every 5 minutes.
**Data source location**	Plitvice Lakes National Park, Croatia (44.8° N, 15.5° E), Dalarna county, Sweden (61° N, 15° E), Hedmark county, Norway (60° N, 11° E)
**Data accessibility**	With the article
**Related research article**	J. Morelli, A. Briganti, B. Fuchs, Ð. Huber, A.L. Evans, S. Reljić, J.M. Arnemo. Comparison of two non-invasive arterial blood pressure monitoring techniques in brown bears (*Ursus arctos*), Veterinary and Animal Science. 9 (2020). https://doi.org/10.1016/j.vas.2020.100094

**Value of the Data**

These data provide baseline values of exertion time, induction time, SAP, DAP, HR, RR, SpO_2_ and Tr in free-ranging brown bears chemically immobilized with xylazine-ketamine after snaring in Croatia or medetomidine-tiletamine-zolazepam after aerial darting in Scandinavia.
These data are useful reference values for assessment of the physiological impacts of capture and chemical immobilization in free-ranging brown bears by veterinarians and other professionals.
These data can be used by researchers to investigate on the different impact on physiology elicited by those two capture and drug protocols, in Croatia and in Scandinavia. The data show that the Korotkoff's technique provided reliable monitoring of BP during immobilization of brown bears in the field. The data needs validation by simultaneous measurements of BP with invasive and Korotkoff's methods.

## 1 Data Description

**Figure 1:**
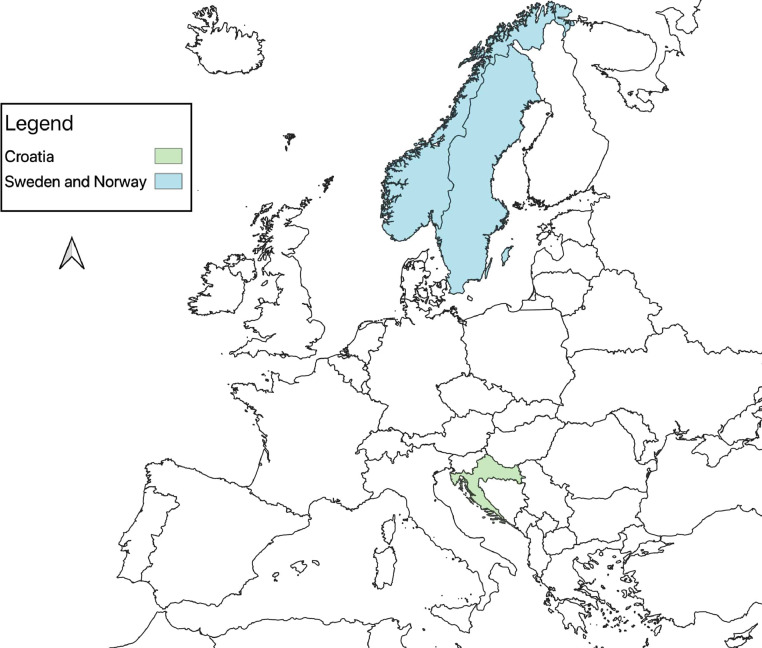
The three countries where 25 chemical immobilizations of brown bears were successfully carried out in this study are highlighted on the map of Europe. When referring to Sweden and Norway, the term “Scandinavia” is adopted in this article .

**Figure 2:**
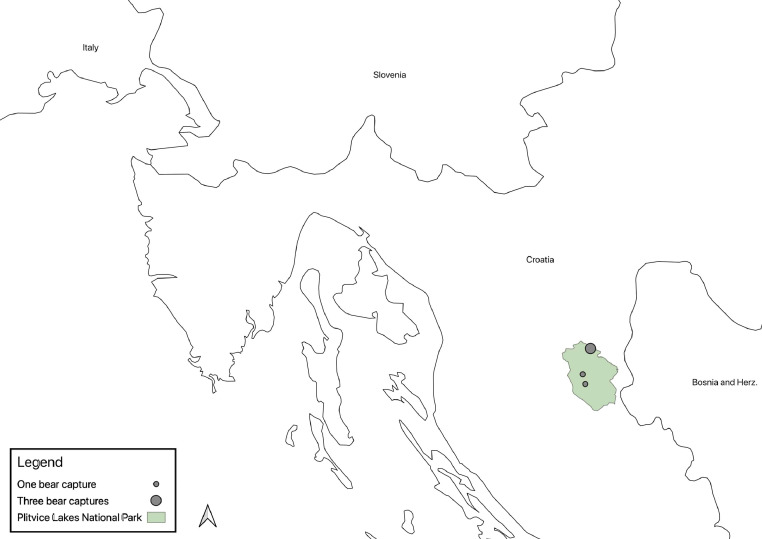
The study area of the captures undertaken in Croatia corresponds to Plitvice Lakes National Park and it is highlighted in the map. Three out of five bears were captured at the same trap site, as denoted by the larger circle on the map.

**Figure 3:**
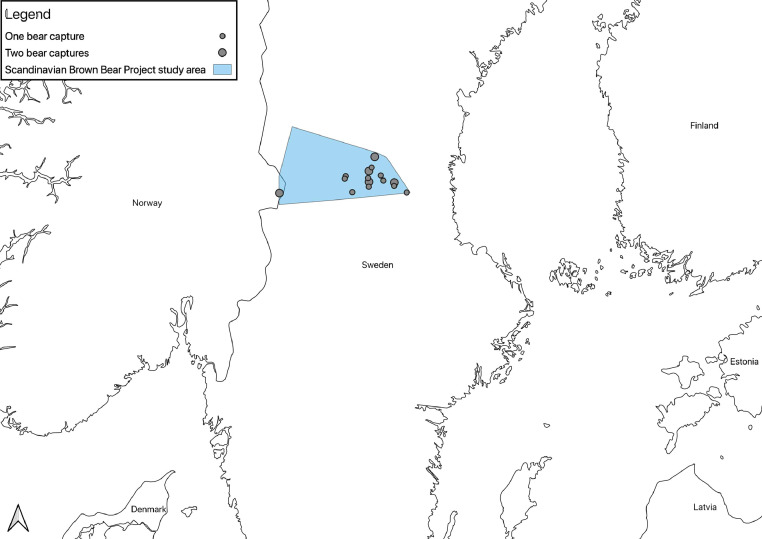
The study area of the Scandinavian Brown Bear Research Project is highlighted on the map of Sweden and Norway. Two out of 20 bears were captured in Norway. At five locations, two bears were captured at the same site, as denoted by larger circles on the map.

**Figure 4:**
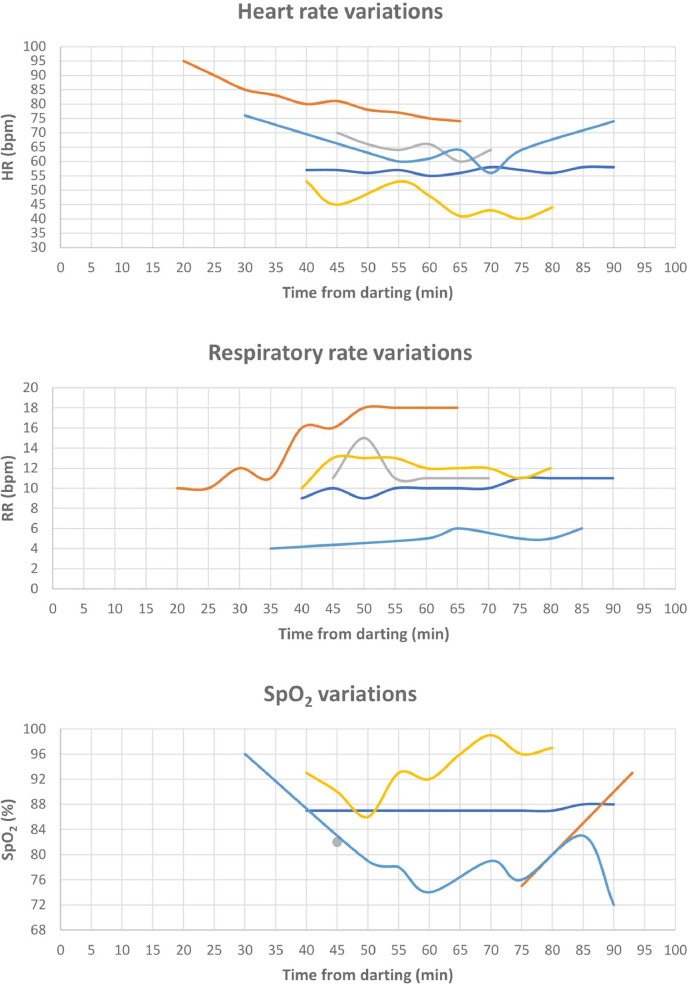
Trends of variation of heart rate (HR), respiratory rate (RR) and hemoglobin-oxygen saturation (SpO_2_) for each bear chemically immobilized in Croatia. Each bear is represented by a different line and time from darting is expressed in minutes and is disposed on the X-axis in every graph. * SpO_2_ of one bear was possible to be measured only once, thus it is presented as a single value rather than a trend of variation.

**Figure 5:**
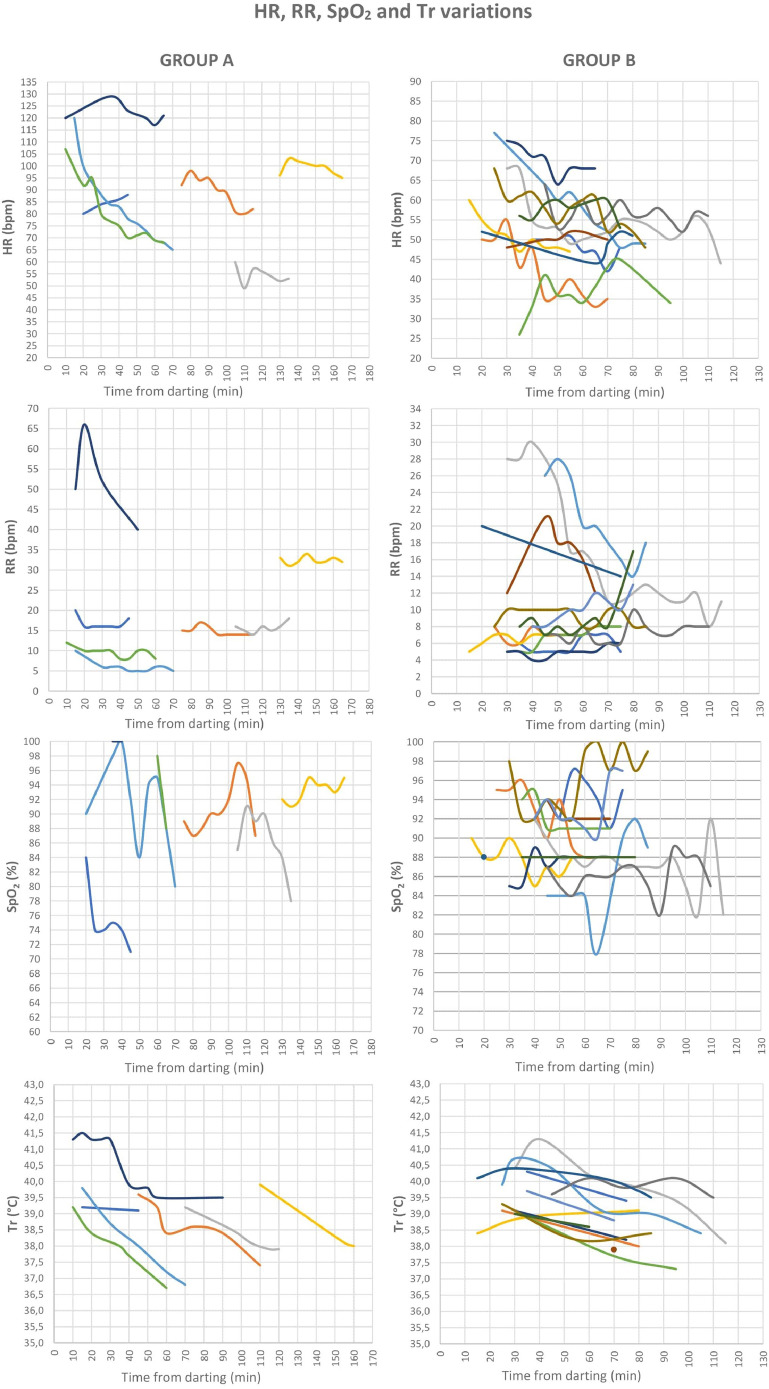
Trends of variation of heart rate (HR), respiratory rate (RR), hemoglobin-oxygen saturation (SpO_2_) and rectal temperature (Tr) for each bear within the groups, chemically immobilized in Scandinavia. Each bear is represented by a different line and time from darting is expressed in minutes and it is disposed on the X-axis in every graph. * SpO_2_ of one bear in Group B was possible to be measured only once, thus it is presented as a single value rather than a trend of variation. * Tr of one bear in Group B was possible to be measured only once, thus it is presented as a single value rather than a trend of variation.

**Figure 6:**
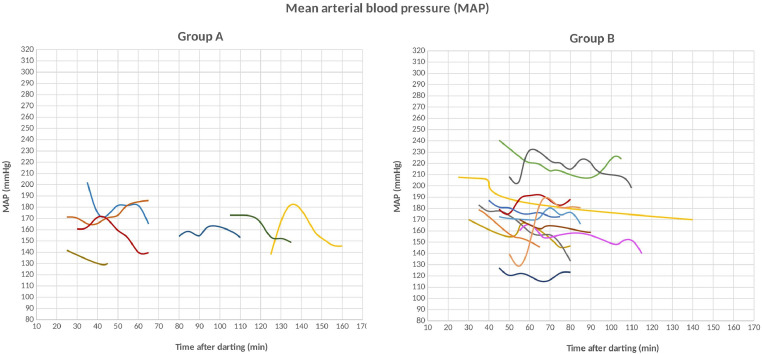
Trends of variation of corrected mean arterial blood pressure (MAP) in 19 bears within the groups, chemically immobilized in Scandinavia. Each bear is represented by a different line and time from darting is expressed in minutes and it is disposed on the X-axis in every graph.

Table 1 presents age, sex, weight of the bears and means (±SD) of all physiological values recorded during the captures for 25 anesthetic events in Croatia (n=5) and Scandinavia (n=20): SAP, DAP, mean arterial blood pressure (MAP), HR, RR, SpO_2_, and Tr. SAP and DAP values are corrected depending on the limb circumference and other bias described below and in recent studies [1], MAP was calculated from SAP and DAP. The ratio between cuff width and limb circumference (CW/LC) ranged from 0.43 to 0.74 in Croatia, and from 0.41 to 0.87 in Scandinavia. Thereby, SAP and DAP were corrected adding from 1.8 mmHg to 11.8 mmHg, and from 1.1 mmHg to 14.0 mmHg to the measured values in Croatia and Scandinavia, respectively. Data on age, sex, weight of the bears, drug doses, exertion time, induction time, and all physiological values recorded during the captures in a time frame, with time starting from the darting with the first drug dose, are presented in the Supplementary File. Figure 1, Figure 2, Figure 3 show the geographic locations of the bear captures, in Croatia and in Scandinavia. Trends of variation of HR, RR, SpO_2_ and Tr of the bears captured in Croatia and in Scandinavia are given in Figure 4, Figure 5, respectively. Figure 6 describes the trends of variation of MAP in the bears immobilized in Scandinavia.

**Table 1:** Age, sex, weight, mean ± SD and range values of systolic arterial pressure (SAP), mean arterial pressure (MAP), diastolic arterial pressure (DAP), heart rate (HR), respiratory rate (RR), hemoglobin-oxygen saturation (SpO_2_) and rectal temperature (Tr) for each bear and within groups (TOT.) throughout 25 chemical immobilizations in Croatia and in Scandinavia. Two bears were excluded (excl.) from the analysis of the blood pressure values.

CROATIA			SAP (mmHg)			MAP (mmHg)			DAP (mmHg)			HR (bpm)			RR (bpm)			SpO_2_ (%)			Tr (°C)			
Age	**Sex**	**Weight**	**Mean**	**± SD**	**Range**	**Mean**	**± SD**	**Range**	**Mean**	**± SD**	**Range**	**Mean**	**± SD**	**Range**	**Mean**	**± SD**	**Range**	**Mean**	**± SD**	**Range**	**Mean**	**± SD**	**Range**	
6	M	109	185	0.00	185*	144	0.00	144*	123	0.00	123*	57	0.98	56-58	10	0.75	9-11	87	0.40	87-88	39.3	0.00	39.3*	
1	M	39	163	10.50	153-181	130	12.31	113-143	113	14.36	94-125	82	6.68	74-95	15	3.53	10-18	85	7.44	75-93	38.6	0.00	38.6	
8⁎⁎	M⁎⁎	189⁎⁎	171	5.50	162-173	139	6.43	130-146	123	7.37	114-132	65	3.29	64-70	12	1.63	11-15	82	0.00	82	38.5	0.00	38.5*	
8⁎⁎	M⁎⁎	176⁎⁎	243	17.03	218-268	178	13.54	157-193	145	14.83	126-162	46	5.03	40-53	12	1.00	10-13	94	3.97	86-99	37.7	0.00	37.7*	
9	F	101	excl.	excl.	excl.	excl.	excl.	excl.	excl.	excl.	excl.	65	6.86	56-76	5	1.00	4-6	80	7.42	72-96	38.7	0.71	38.2-39.2	
TOT.			196	11.04	153-268	151	11.84	113-193	128	11.89	94-162	63	4.47	40-95	12	1.68	4-18	86	4.02	72-99	38.6	0.20	37.7-39.3	

SCANDINAVIA				**SAP (mmHg)**			**MAP (mmHg)**			**DAP (mmHg)**			**HR (bpm)**			**RR (bpm)**			**SpO_2_ (%)**			**Tr (°C)**		
Group	**Age**	**Sex**	**Weight**	**Mean**	**± SD**	**Range**	**Mean**	**± SD**	**Range**	**Mean**	**± SD**	**Range**	**Mean**	**± SD**	**Range**	**Mean**	**± SD**	**Range**	**Mean**	**± SD**	**Range**	**Mean**	**± SD**	**Range**
A	1	F	18	excl.	excl.	excl.	excl.	excl.	excl.	excl.	excl.	excl.	84	2.86	80-88	17	1.57	16-20	75	4.46	71-84	39.2	0.07	39.1-39.2
	1	F	22	179	2.88	176-184	157	3.80	153-163	146	6.34	139-155	89	6.58	80-98	15	1.09	14-17	91	3.50	87-97	38.6	0.76	37.4-39.6
	1	F	24	194	9.39	180-203	163	10.83	149-173	147	11.75	133-158	54	3.60	49-60	16	1.25	14-18	86	4.45	78-91	38.3	0.55	37.9-39.2
	1	M	21	178	19.18	158-213	155	15.85	138-182	143	14.26	128-167	99	2.92	95-103	32	0.92	31-34	93	1.49	91-95	38.8	0.90	38.0-39.9
	1	F	18	175	13.66	155-189	157	11.94	140-171	148	11.26	132-162	82	16.86	65-120	5	1.62	5-10	91	6.48	80-100	38.1	1.20	36.8-39.8
	1	F	19	204	7.41	197-219	173	7.35	165-186	158	7.43	150-170	80	12.81	68-107	10	1.27	8-12	93	7.07	88-98	38.0	0.92	36.7-39.2
	1	F	18	208	11.76	198-233	180	11.46	165-202	166	11.94	148-187	122	4.08	117-129	52	10.71	40-66	100	0.00	100	40.6	0.89	39.5-41.5
TOT. A				190	10.69	155-233	164	10.20	138-202	152	10.45	128-187	86	8.20	49-129	18	1.98	5-66	89	4.04	71-100	39.0	0.82	36.7-41.5
B	4	F	59	197	26.11	157-217	166	23.41	129-188	148	27.34	105-174	58	2.54	53-60	9	3.08	7-17	88	0.00	88	38.8	0.28	38.6-39.0
	3	F	56	227	12.11	208-240	186	5.86	175-192	165	4.02	156-168	59	2.69	55-64	10	1.69	8-13	93	2.64	90-97	39.3	0.64	38.8-39.7
	6	F	83	215	4.59	208-221	178	4.90	173-187	159	7.33	150-170	48	2.90	42-51	6	0.97	5-7	94	2.32	91-97	39.9	0.64	39.4-40.3
	5	F	61	203	7.53	194-214	160	13.09	146-179	138	15.94	121-161	42	7.67	33-55	7	0.79	6-8	92	3.31	88-96	38.6	0.57	38.0-39.1
	12	F	86	209	9.84	191-224	154	7.44	140-166	127	7.62	114-140	54	5.86	44-68	16	7.24	8-30	87	2.84	82-92	40.0	1.25	38.1-40.2
	24	F	90	266	43.89	191-298	195	16.28	170-208	159	5.87	149-164	51	4.31	57-60	7	0.73	5-7	88	1.64	85-90	38.7	0.35	38.4-39.1
	8	F	97	268	5.30	258-273	173	4.33	166-180	125	4.92	120-135	57	8.98	48-77	21	4.90	14-28	86	4.47	78-92	39.6	0.72	38.4-40.7
	17	F	71	294	15.22	269-319	221	9.61	207-241	184	7.45	176-201	37	5.50	26-45	7	1.17	5-8	92	1.64	91-94	38.0	0.83	37.3-38.9
	16	F	123	169	15.26	154-196	121	3.75	116-127	97	6.36	93-108	70	3.60	64-75	5	0.67	4-6	87	1.49	85-89	38.7	0.64	38.2-39.1
	6	M	154	219	3.71	216-225	163	4.41	159-170	135	6.40	130-146	50	1.40	48-52	16	3.60	12-21	92	0.82	92-94	37.9	0.00	37.9
	8	M	241	296	5.84	285-305	215	9.70	198-232	174	13.06	155-195	57	3.13	52-64	7	1.14	6-10	86	1.82	84-89	39.8	0.28	39.5-40.1
	8	F	87	244	23.98	203-281	163	15.54	133-183	123	11.89	98-134	58	5.35	48-68	9	1.01	8-10	96	3.26	92-100	38.6	0.59	38.2-39.3
	12	F	83	218	11.03	202-232	157	9.58	145-170	127	9.76	117-139	50	3.36	44-52	17	4.24	14-20	88	0.00	88	40.0	0.36	39.5-40.4
TOT. B				235	13.69	154-319	175	9.83	116-241	144	10.03	93-201	53	4.66	26-77	10	2.46	4-28	90	2.22	78-100	39.3	0.61	37.3-40.7

⁎ assessed in only one 5-minute gap.

⁎⁎ this bear was trapped twice in this study.

## 2 Experimental Design, Materials, and Methods

### 2.1 Study area and animals

The study included five chemical immobilizations of four free-ranging brown bears in Plitvice Lakes National Park, Croatia (44.8° N, 15.5° E; 300-800 m above sea level), as part of an ongoing research project. Three adult bears (one female and two males - one male was captured twice), and one male yearling (1-year-old) were trapped in April and May 2016, during the night and early morning (between 7 p.m. and 6 a.m.) with ambient temperatures (T_a_) ranging from 5 °C to 16 °C.

In the second part of the study, 20 free-ranging brown bears were chemically immobilized in the counties of Dalarna, Sweden (61° N, 15° E) and Hedmark, Norway (60° N, 11° E; 300-700 m above sea level), in the scope of the Scandinavian Brown Bear Research Project's ongoing research. The captures took place in April and May 2017, during the day (9 a.m. to 7 p.m.). T_a_ ranged from 4 °C to 22 °C. The Scandinavian bears were divided into two groups based on age: Group A consisted of yearlings (n = 7), whereas group B consisted of subadults [2] (2-4 years old, n = 2) and adults (n = 11).

### 2.2 Capture methods, drugs and darting materials

In Croatia, bears were trapped with Aldrich spring-activated foot snares equipped with a GSM-based alarm. The bears were darted with a combination of xylazine (Rompun®, Bayer AG, Leverkusen, Germany, 500 mg dry substance) and ketamine (Ketaminol® 10, Intervet AB, Stockholm, Sweden, 100 mg/ml) (XK). The drugs were mixed by adding 5ml of ketamine to xylazine dry substance so that 1ml of the solution contained 90.9 mg xylazine and 90.9 mg of ketamine and administered with a CO_2_ powered rifle (Dan-Inject®, Børkop, Denmark). Additional XK was administered after 15 minutes if the bear was not immobilized. Before commencement of the procedures, 3-5 mg/kg of ketamine was administered intramuscularly (IM) by hand syringe depending on the anesthetic plane. Drug doses are reported in the Supplementary File and by Morelli et al. [1]. Bears were equipped with global positioning system (GPS) radio-collar (Vectronic Aerospace®, Berlin, Germany) and were left to recover close to the trap site. All bears recovered completely (confirmed by camera-traps and GPS positioning).

In Scandinavia, bears were located through radiotracking and darted from a helicopter with a CO_2_ powered rifle (Dan-Inject®). The time of intensive helicopter pursuit did not exceed 1 minute. Scandinavian bears were darted with a combination of medetomidine (Domitor®, Orion Pharma, Espoo, Finland, 1 mg/ml, or Zalopine®, Orion Pharma, Espoo, Finland, 10 mg/ml) and tiletamine-zolazepam (Zoletil Forte® Vet, Virbac, Carros, France, 50 mg/ml) (MTZ) according previously established protocols [3]. At the end of the procedures, the effects of medetomidine was antagonized with atipamezole (Antisedan®, Orion Pharma, Espoo, Finland, 5 mg/ml), administered IM at five times the total dose of medetomidine and the bears were left to recover at the site of capture. For 30 days after captures, the activity of all bears were monitored by telemetry and successful recovery was confirmed in all cases. All Scandinavian bears were moved to dorsal recumbency and underwent abdominal surgery in order to implant or retrieve Very High Frequency (VHF) transmitters or bio-loggers [3], and both subadult and adult bears were also equipped with GPS radio-collars (Vectronic Aerospace®). Analgesia was provided by administering meloxicam subcutaneously at 0.4 mg/kg (Metacam®, Boehringer Ingelheim, Ingelheim , Germany, 20 mg/ml).

### 2.3 Monitoring

Rectal temperature was measured as soon as possible after recumbency when approach was considered safe with a digital thermometer and then monitored at frequent intervals (varied between individuals) during the immobilization. Anesthetic depth was assessed by evaluating palpebral reflex, jaw tone and positioning of the eyeball every 5 minutes. In order to maintain an adequate level of anesthesia, additional ketamine (2-3 mg/kg) was given IM, if required. Heart rate and hemoglobin-oxygen saturation (SpO_2_) were measured by pulse oximetry (OxyVet, Eickemeyer Veterinary Equipment Ltd, Sunbury-on-Thames, UK and NPB-40, Infiniti Medical®, Menlo Park, USA in Croatia and in Scandinavia, respectively) every 5 minutes. The pulse oximeter probe was attached to the tongue and the readings for HR were consistent with the rate detected with a stethoscope (Littmann® Classic II S.E., 3M®, USA). Respiratory rate was monitored every 5 minutes by either using a stethoscope or counting the thoracic wall excursions over 30 seconds. The aforementioned variables were recorded at frequent intervals (varied between individuals) in a limited number of bears, as presented in the Supplementary File. This discrepancy from the initial plan was due to technical difficulties and to the main focus on BP monitoring in the original study [1]. A standard aneroid sphygmomanometer (DM330LF, LOGIKO VISUAL®, Moretti S.p.a. Italy; pressure range 0-300 mmHg, accuracy of ± 2 mmHg), which consisted of a manometer, a cuff, a valve and a balloon, was used to monitor systolic and diastolic arterial blood pressure (SAP and DAP, respectively). The instrument was purchased immediately before the first day of captures and was calibrated with a mercury manometer every six months until the last day of the BP monitoring. The hair proximal to either the carpus or the tarsus was clipped, the limb circumference was measured and the ratio between the cuff width and the limb circumference (CW/LC) was calculated. The manual technique detected Korotkoff's sounds of the pulse (so SAP and DAP, corresponding to phase I and phase V, respectively) through the diaphragm of a stethoscope placed above the medial plantar artery, distally from the cuff. Then, MAP was calculated from SAP and DAP values. SAP and DAP were measured continuously and simultaneously with both techniques with the same operator, in order to obtain from one to three values every 5 minutes from each device. Thus, the mean values for SAP, DAP, and MAP were calculated within every 5-minute interval. SAP and DAP were measured as long as possible during the procedures in all bears from single captures (n = 5 in Croatia, n = 7 in Scandinavia) and for 30 minutes per individual in case of family captures (n = 13). In case of suspected malfunction of the automatic sphygmomanometer, either a solution of soap and water was dispensed between the limb and the cuff in order to improve adherence and the reading, or the cuff was changed. SAP and DAP values were corrected in relation to the size of the applied cuff using derived calculations from a study on different cuff sizes used in human patients [4]. These corrective formulae consider a CW/LC of 0.41 as optimal. In this way, we reduced the bias of the different CW/LC_,_ though with the assumption of a similar correlation between CW/LC and overestimation (or underestimation) of SAP and DAP in bears. Thus, the corrective formulae applied to SAP and DAP values for the sphygmomanometer's cuff (14 cm-wide) are, respectively:

SAPc=SAPm+26.2−0.76×LC

DAPc=DAPm+16.9−0.49×LC

where the subscripts “c” and “m” indicate the corrected value and the measured value, respectively, and LC is the limb circumference. These coefficients have been extrapolated from the trendlines of the original coefficients [4].

SAP and DAP values were also corrected decreasing or increasing by 0.8 mmHg for each cm of the vertical distance between the inflated cuff and the right atrium level, whenever the cuff was placed more than 10 cm below or above, respectively.

## CRediT authorship contribution statement

**Jacopo Morelli:** Conceptualization, Methodology, Formal analysis, Investigation, Writing - original draft, Visualization, Supervision, Funding acquisition. **Angela Briganti:** Conceptualization, Methodology, Resources, Writing - review & editing. **Boris Fuchs:** Methodology, Software, Formal analysis, Writing - review & editing. **Ðuro Huber:** Validation, Resources, Writing - review & editing, Supervision, Project administration, Funding acquisition. **Alina L. Evans:** Methodology, Validation, Resources, Writing - review & editing, Supervision, Project administration. **Natarsha Babic:** Software, Formal analysis, Writing - review & editing, Visualization. **Slaven Reljić:** Resources, Writing - review & editing, Visualization, Supervision, Project administration. **Lana Pađen:** Resources, Writing - review & editing, Supervision. **Jon M. Arnemo:** Methodology, Validation, Resources, Writing - review & editing, Supervision, Project administration, Funding acquisition.

## Declaration of Competing Interest

The authors declare that they have no known competing financial interests or personal relationships which have, or could be perceived to have, influenced the work reported in this article.

## Appendix Supplementary materials

Image, application 1

Image, application 2

Image, application 3
