# Supplementary material for: NON-INVASIVE BLOOD PRESSURE AND OTHER PHYSIOLOGICAL DATA IN CHEMICALLY IMMOBILIZED BROWN BEARS (URSUS ARCTOS)
Source: Data Brief. 2020 Apr 29;30:105646. doi: 10.1016/j.dib.2020.105646 (PMC7214828; doi:10.1016/j.dib.2020.105646)
Supplement: Supplementary file 3 [file mmc3.docx]

**Supplementary File:** Age, sex, weight, drug dose, exertion time, induction time and values of systolic arterial pressure (SAP), mean arterial pressure (MAP), diastolic arterial pressure (DAP), heart rate (HR), respiratory rate (RR), hemoglobin-oxygen saturation (SpO2) and rectal temperature (Tr) for each bear throughout 25 chemical immobilizations in Croatia (n=5) and in Scandinavia (n=20). The Scandinavian bears were divided in group A (yearlings, n=7) and group B (subadults and adults, n=13). Exertion time is expressed in minutes (min) and is considered to be from when the trap was triggered (Croatia) or from the start of the helicopter chase (Scandinavia) to the recumbency. Induction time is expressed in minutes (min) and is considered to be from the time between darting to the recumbency. Time zero corresponds to the time of darting with the anesthetic drug combination. SAP, MAP and DAP values are corrected depending on different biases (e.g. cuff width:limb circumference ratio) and represent mean values of 2-4 measurements carried out in each 5-minutes gap. Two bears were excluded (excl.) from the analysis of the blood pressure values.

* medetomidine (M) is expressed in μg/kg. Xylazine (X), ketamine (K) and tiletamine-zolazepam (TZ) are expressed in mg/kg.

** this bear was trapped twice in this study.

M: male

F: female

SAP: systolic arterial pressure

MAP: mean arterial pressure

DAP: diastolic arterial pressure

HR: heart rate

RR: respiratory rate

SpO2: hemoglobin oxygen saturation

Tr: rectal temperature
